# Development of a Wireless Unified-Maintenance System for the Structural Health Monitoring of Civil Structures

**DOI:** 10.3390/s18051485

**Published:** 2018-05-09

**Authors:** Gwanghee Heo, Byungjik Son, Chunggil Kim, Seunggon Jeon, Joonryong Jeon

**Affiliations:** 1 Department of International Civil and Plant Engineering, Konyang University, 121 Daehak-ro, Nonsan, Chungnam 32992, Korea; heo@konyang.ac.kr (G.H.); strustar@konyang.ac.kr (B.S.); cg-kim@konyang.ac.kr (C.K.); 2 Department of Civil Engineering, Chungnam National University, Daejeon 34134, Korea; gonylife@hanmail.net

**Keywords:** wireless unified-maintenance system, wireless sensor networks, modal characteristics, finite element analysis, modal test, civil structures, structural health monitoring

## Abstract

A disaster preventive structural health monitoring (SHM) system needs to be equipped with the following abilities: First, it should be able to simultaneously measure diverse types of data (e.g., displacement, velocity, acceleration, strain, load, temperature, humidity, etc.) for accurate diagnosis. Second, it also requires standalone power supply to guarantee its immediate response in crisis (e.g., sudden interruption of normal AC power in disaster situations). Furthermore, it should be capable of prompt processing and realtime wireless communication of a huge amount of data. Therefore, this study is aimed at developing a wireless unified-maintenance system (WUMS) that would satisfy all the requirements for a disaster preventive SHM system of civil structures. The WUMS is designed to measure diverse types of structural responses in realtime based on wireless communication, allowing users to selectively use WiFi RF band and finally working in standalone mode by means of the field-programmable gate array (FPGA) technology. To verify its performance, the following tests were performed: (i) A test to see how far communication is possible in open field, (ii) a test on a shaker to see how accurate responses are, (iii) a modal test on a bridge to see how exactly characteristic real-time dynamic responses are of structures. The test results proved that the WUMS was able to secure stable communication far up to nearly 800 m away by acquiring wireless responses in realtime accurately, when compared to the displacement and acceleration responses which were acquired through wired communication. The analysis of dynamic characteristics also showed that the wireless acceleration responses in real-time represented satisfactorily the dynamic properties of structures. Therefore, the WUMS is proved valid as a SHM, and its outstanding performance is also proven.

## 1. Introduction

It is important all civil structures are securely built and maintained from the very beginning until the end of their life since their insecurity may endanger human lives and public well-being. However, it is not easy to secure their safety because they are constantly exposed to deterioration due to aging, excessive load, and carelessness [1,2,3], and also natural disasters as well, thus threatening public safety. Recently, many catastrophic natural disasters took place such as massive earthquakes, tsunamis, and storms all around the world, in the U.S., Italy, India, Japan, China, Taiwan and Mexico, for instance. When buildings become particularly higher, larger and denser these days, they are likely to be in even greater danger than before. Thus, it is critical to have a structural health monitoring (SHM) system to examine building conditions regularly [4,5]. SHM technologies detect structural damages or dangers by means of the response information from buildings, significantly saving the time and efforts (e.g., repairs and reinforcements) [6,7]. Therefore, there have been a number of studies on SHM to secure a safe and effective maintenance of buildings [7,8,9,10].

In the past, SHM systems were developed with wired sensors and centralized processing [4]. However, such conventional SHM systems had some difficulties in supplying power directly to wired sensors, and they also had too much noise and cost for cabling for the purpose of transmitting data from wired sensors to data loggers. To solve those problems, there have been many studies on wireless measurement systems [11,12]. Straser et al. (1998) [13] proposed a wireless measurement system for the first time to replace the wired system. The early-stage wireless measurement system proposed by Straser was a simple system which sent the data in real-time to a single concentrated data logger after obtaining them from the sensors attached to the buildings. Later, Spencer et al. (2004) [14] and Lynch et al. (2006) [15] introduced the wireless SHM systems (e.g., MICA, iMote series, etc.) to the construction industry, using the hardware platform which was upgraded to a SHM-enabled level. Rice et al. (2008) [16] and Nagayama et al. (2009) [17] carried out tests to verify the performances of iMote series (calibration testing, noise and resolution, clock accuracy, power consumption) and modal tests using model truss. Jang et al. (2010) [18] also performed a test in which they obtained responses for each bridge member (e.g., tower, superstructure, cable, etc.) in real-time by applying a total of 70 iMote2 units to the second Jindo cable-stayed bridge (Republic of Korea) [18]. Then, the validity of the developed wireless sensor node was confirmed from the results of these experimental studies, suggesting that the wireless measurement system could be used to secure structural integrity under accidental risky circumstances.

Despite all those advantages, however, the wireless SHM systems were not able to easily adjust their hardware and software to the variable site conditions because they were designed to work under limited conditions in terms of wireless communication distance, number of channels, size of the acquired data, etc. Furthermore, their sensor nodes were poorly equipped with a micro-controller whose CPU performance was low and whose memory was limited (e.g., Atmel ATmega 128(L), Intel XScalePXA271) only to reduce costs and power by making it smaller in size and weight. Thus, they were limited in acquiring and processing a large amount of consecutive signals in real-time [19,20,21]. Although wireless communication technologies have been far advanced these days, producing some new wireless sensor nodes, their standardized user platform (hardware, software) still fails to take into account variable site conditions. That is, wireless SHM system developed so far are still limited in actual performance [22,23,24].

To overcome such problems, therefore, this study has developed a wireless unified-maintenance system (WUMS). The WUMS is designed to be applicable to real structures performing the following specific functions. (1) Its variable sensor input and output (I/O) makes realtime multi-sensing possible, taking into account measuring conditions (e.g., measured signal type, number of measurement channels, etc.) and it also enables an assessment of structural performance by making measured signals synchronized by channel. (2) Its program is to effectively acquire and process a large amount of signals in real-time by means of a micro-processor equipped with a high performance CPU (400 MHz) and a 256 MB flash memory (128 MB system memory). (3) The algorithms embedded in it make it possible to acquire, transmit, receive signals wirelessly by means of advanced field-programmable gate array (FPGA)-based embedded-software technology (EST) technology and real-time operating system (RTOS), and they also allow the system to be operated in standalone mode. (4) It enables stable and fast wireless communication by freely selecting the optimum communication frequency (2.4 GHz or 5 GHz) in accordance with the communication environment of buildings by means of the WiFi dual-band wireless access point (AP). (5) The system is also designed to have its own power source for stable power supply for a certain period of time (approximately 48 h) with 5200 mAh battery. Based on user needs and measurement environments stated above, the WUMS is to measure diverse responses from buildings (e.g., displacement, velocity, acceleration, strain, etc.) in realtime and perform an accurate analysis and assessment.

## 2. The Design of the Wireless Unified-Maintenance System

### 2.1. Hardware Design of the WUMS

**Figure 1:**
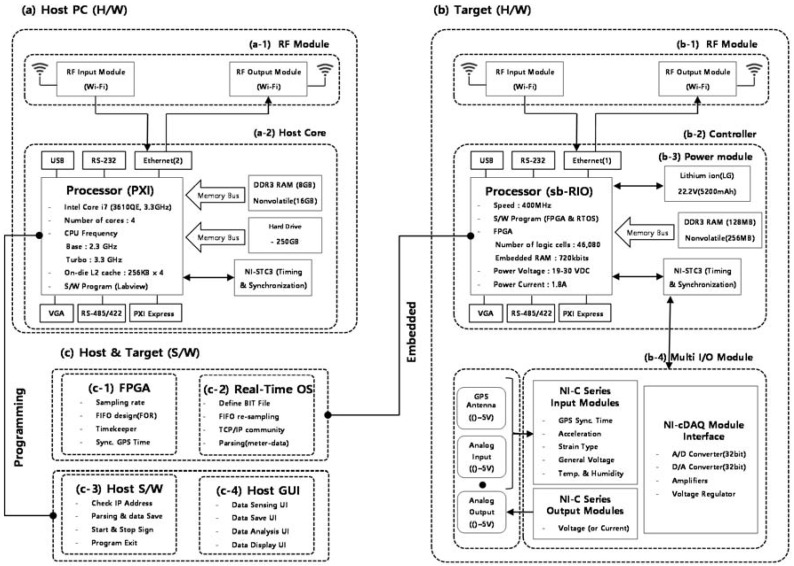
Wireless unified-maintenance system (WUMS): (**a**) Host PC; (**b**) target; (**c**) host and target S/W.

The WUMS was developed in two different categories as shown in Figure 1 below: Hardware (Figure 1a,b) and software (Figure 1c). First, the hardware was developed into a host PC (Figure 1a) and a target (Figure 1b) which includes controller, GPS module, response measurement model, RF module and power module. Here, the host PC functions as a monitor that receives and controls the structural responses measured and transmitted from the target. The target, installed in actual structures, plays a role of measuring diverse structural responses and delivering them to the host PC. 

**Figure 2:**
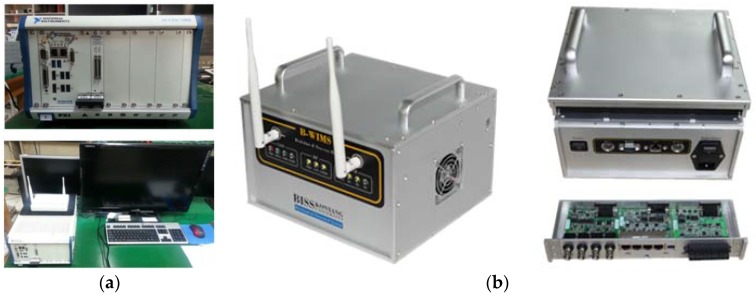
Component of H/W of the WUMS: (**a**) Host PC; (**b**) target.

**Figure 3:**
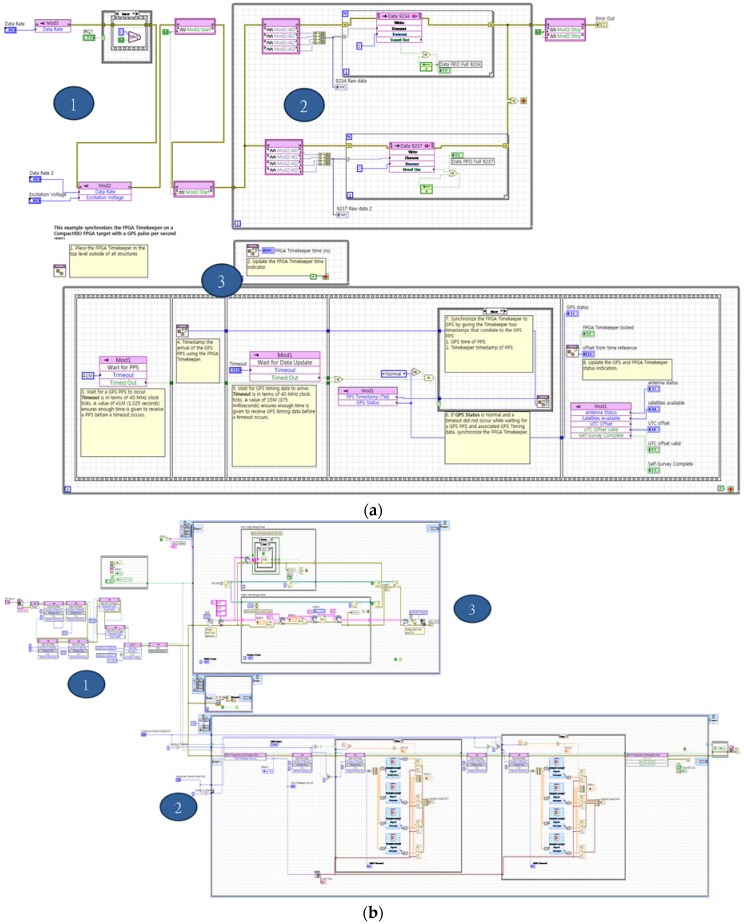
Target S/W for wireless unified-maintenance system (WUMS): (**a**) field-programmable gate array (FPGA); (**b**) real-time operating system (RTOS).

**Figure 4:**
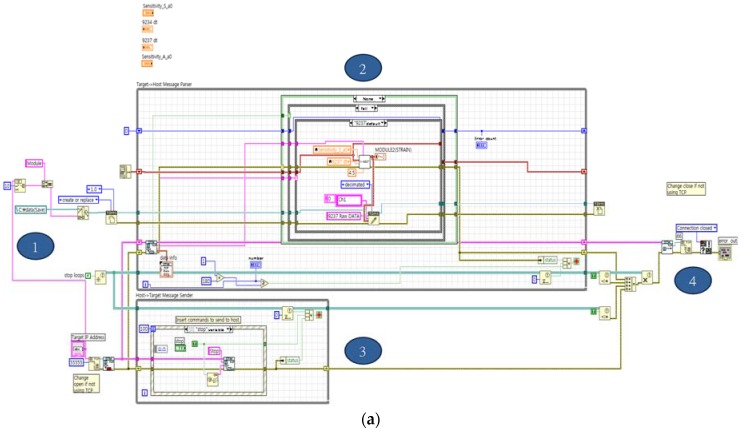

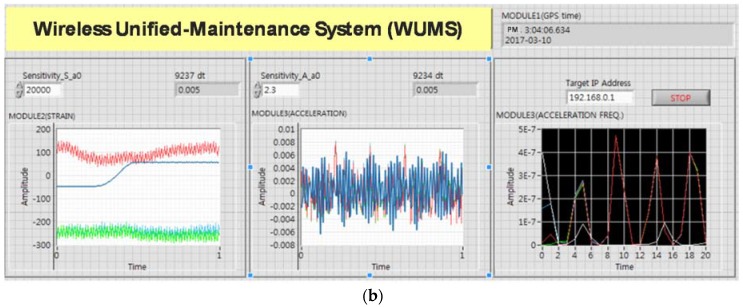
Host S/W for Wireless unified-maintenance system (WUMS): (**a**) Host; (**b**) graphical user interface (GUI).

First, in this study, the WUMS adopted a NI sbRIO-9602 that provides three slots so as to be connected with multiple external sensors, as shown in Figure 1(b–2) for the target controller which constituted the hardware of the WUMS. Its adoption made it possible to design a multiple I/O which helped effectively measure diverse types of structural responses, which is necessary to develop a SHM of buildings. In addition, it also employed a high-performance CPU and an internal memory both of which resulted in a remarkable improvement in its ability to handle in realtime diverse types of structural response obtained from the multi I/O module as in Figure 1(b–4). Furthermore, its use of the FPGA and RTOS, which are operable in NI sbRIO-9602, facilitated a secure embedding of the logics into a controller in regard to measurement, processing and transmission of structural response, as shown in Figure 1(c–1,c–2). In the meantime, its controller was to work on the basis of the embedded logics so that the target would be operated in real-time standalone mode. It is also important to stabilize power supply for the smooth operation of the controller during regular and irregular times. For this, the WUMS in this study adopted a LG Li-ion battery as shown in Figure 1(b–3) so as to supply power steadily to the target controller even in case of emergencies. The LG Li-ion battery in Figure 1(b–3) is able to generate 22.5 V and 5200 mAh with twelve 3.7 V batteries when connected in series and parallel. In this study, its adoptation in the target ensured internal power supply for about 48 hours without external power insertion.

Then, a multi I/O (Figure 1(b–4)) was designed on each of the three slots of the controller to measure GPS time, acceleration response, and strain response. In order to measure GPS time, a NI-9467 was used on the first slot. It is a GPS module specially designed for the NI-sbRIO series as a controller; it is to measure GPS time by linking it to a separate GPS antenna of a magnetic type. In this study, the target’s reference time was set on GPS time by using the NI-9467. The multi I/O on the first slot was also designed to synchronize each different measurement time in accordance with each different target when several targets were used. Next, to measure the acceleration response of structures, a NI-9234 was adopted. It is able to get responses from the Piezo-type acceleration sensors by means of four channels. Each channel has an antialiasing filter which is automatically adjusted to each different sampling speed, with a capacity to measure the responses at the maximum speed of 51.2 kHz per channel. On the second slot was a multi I/O designed to get the acceleration responses required for a SHM at high speed, using the NI-9234 without any signal distortion. Then, the NI-9237 module was used on the third slot to measure strain response. The NI-9237 is able to get diverse responses from strain-type sensors (e.g., displacement, load, slope, etc.) through four channels, and each channel has a 24-bit synchronous bridge. The responses can be measured at up to 50 kHz per channel. This multi I/O on the third slot is able to get the multiple responses bridged simultaneously.

While composing the hardware of the WUMS, this study adopted a NI PXIe-8135 for the host PC as shown in Figure 1a. The NI PXIe-8135 is a high-performance PC with a 2.3 GHz quad core (Intel Core i7-3610QE) processor and a 1600 MHz DDR3 dual memory. It enabled the WUMS to efficiently operate, process and save the response signals received from the target in realtime. Then, the logic and the graphical user interface (GUI) were programmed in accordance with the operation particular to the WUMS as shown in Figure 1(c–3,c–4).

To enable two-way wireless communication between the host PC and the target, lastly, a RF module was adopted as shown in Figure 1(a–1,b–1). The RF ipTIME-A2004NS-R module is sensitive enough to receive data up to 867 Mbps at maximum, based on WiFi. In it, the two-way wireless communication can be selectively chosen at WiFi 2.4 GHz or 5 GHz RF band. In this study, a ipTIME-A2004NS-R was adopted so as to make it possible to select freely RF bands in accordance with the site conditions of structures (e.g., target installation location, communication obstacle, etc.). Then, a two-way RF module was designed to get structural responses effectively and output the host commands (e.g., realtime feedback control) if necessary. The hardware (host PC and target) developed from the selection of the said diverse modules and organic hardware configuration are shown in Figure 2 below:

### 2.2. Software Design of the WUMS

The software of the WUMS was designed in two different categories as shown in Figure 1c: target software and host PC software. First, the target software (Figure 1(c–1,c–2)) is supposed to obtain the responses from sensors and transmit them. To make the WUMS work in a standalone mode, each of the FPGA (Figure 3a) and the RTOS (Figure 3b) was separately programmed. 

First, the FPGA program in Figure 3a was developed under the following three main logics: (1) It is designed to decide on the sampling rate of each measurement module (e.g., NI-9467, 9234, 9237) by second and then initiate them. (2) A FIFO (first-in first out) was constructed to get only a single piece of information at a time by each channel by means of the analogue voltage input at each different sampling rate by second in accordance with each module through the repetitive statement (FOR loop). (3) To create GPS values in real-time in the FPGA, the FPGA TIMEKEEPER was used after being programmed to synchronize the signals collected by each module in accordance with the created GPS time interval (1 pps).

In the meantime, the RTOS program in Figure 3b was developed in the following three main logics: (1) To operate the FPGA in the RTOS, the BIT file, created by the FPGA, was defined. Then, all I/O data were defined through timeout of FIFO and maximum readable sampling. In addition, it was designed to initialize each variable and residual data accumulated in the FIFO at the start of the program. (2) For the accurate and efficient data collection by lowering excessive data load increased by wireless transmission and reception, the data collected through the FIFO were re-sampled by referring sampling rates per measurement module decided in the FPGA as in Figure 2a. (3) The collected data were transmitted to the consumer loop through the producer and consumer loop. The consumer loop was to transmit and receive the transferred data along with metadata through the TCP/IP. Here, since they included the information on the transmitted data (e.g., module type, number of channel, data sample rates/s, etc.), metadata were parsed for user convenience. As shown so far, the target software was programmed to be operated in a standalone mode by fully embedding it into the target.

In addition, the software of the host PC (Figure 1(c–3,c–4)) was developed into the two—the operating program (Figure 4a) and GUI (Figure 4b)—to receive, save, analyze and display the response signals received from the target. The operating program in Figure 4a was developed under the following four main logics: (1) After checking the IP address of the target to collect data, the data storage position was decided. (2) Data were collected after classifying the data from the target using metadata. (3) In order to end communication, an event case was parallelized (true or false) to create a stop command. (4) After data collection, an error is forced to occur for an end of communication, eventually for a termination of program. Lastly, the GUI program in Figure 4b was developed to display the results (e.g., measured data by module or channel, analysis results) of the logics developed by the host based on the operating program in Figure 3a.

## 3. Evaluation Experiments of the WUMS

To test the performance of the developed WUMS, this study carried out the following experiments: (i) A test to identify how far communication is possible in an open field, (ii) a response test using a shaker, (iii) a prototype bridge-based modal testing.

### 3.1. Wireless Communication Distance on Open Field

**Figure 5:**
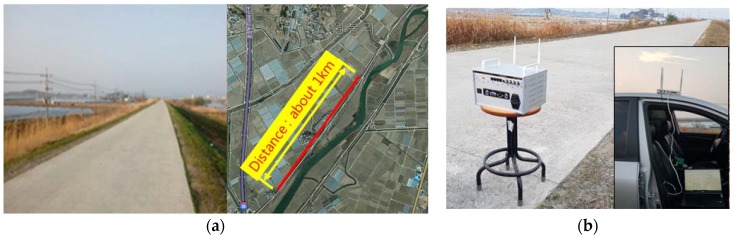
Test of wireless communication distance: (**a**) view of open field; (**b**) test setup in open field.

The previous section informed that an ipTIME-A2004NS-R was adopted for the target’s RF module. The ipTIME-A2004NS-R module enables selective two-way wireless communication at WiFi 2.4 GHz or 5 GHz based on WiFi. In general, the WiFi 2.4 GHz uses a relatively low frequency band, and so its wavelength is large, while its reflection and refraction on an obstacle are far more efficient than that of WiFi 5 GHz. In contrast, the WiFi 5 GHz uses a relatively high frequency band so that its wavelength is small, but its linear signal sensitivity is more efficient than WiFi 2.4 GHz. After all, the RF band can vary depending on the site conditions of target structures and the position of response measurement. This study attempted to test on communication distance by the RF band which can be selectable by the WUMS. For this, we chose to carry out the experiment in the open field in which radio interference was relatively low without any obstacles and linear distance could be secured as shown in Figure 5 below:

For the test of communication distance, a riverside open field in Nonsan city (Nonsan-si, Chungnam, South Korea) was chosen because it had over 1 km straight roadlinear distance with low signal interference. In addition, no obstacles were found. Figure 5a reveals the view of the open field with its satellite photo, and Figure 5b shows the WUMS installed on the open field for the test of communication distance. Each of the tests at WiFi 2.4 GHz was carried out first at the point of 100 m away from the WUMS and then at every 100 m further up to 1 km, and the same tests were carried out at WiFi 5 GHz. All tests were aimed to find how far the WUMS would be sensitive enough to receive communication signals. Its sensitivity is tabled in Table 1 and Table 2 below.

As shown in Table 1, the WUMS was found to have a capacity of receiving signals up to about 500 m at WiFi 2.4 GHz with 70% signal sensitivity. In the meantime, Table 2 shows it was able to grasp signals, this time, up to 800 m at WiFi 5 GHz with 60% signal sensitivity. Considering the fact that most construction buildings are located in the outskirts or open fields, and also the WiFi 5 GHz is found relatively advantageous against signal interference, this study chose the WiFi 5 GHz for the RF band of the WUMS. Then, a shaker-based response test and a prototype bridge-based modal test were performed separately.

### 3.2. Response Tests Using a Modal Shaker

**Figure 6:**
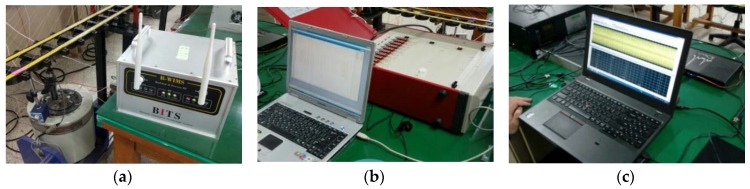
Response test using a modal shaker: (**a**) Wireless responses (displacement and acceleration) test; (**b**) wired response (displacement) test; (**c**) wired response (acceleration) test.

To assess the initial response performance of the WUMS, this study carried out static and dynamic response tests using a modal shaker on both wired and wireless setting. First, displacement and acceleration responses were wirelessly measured at the same time, using both NI-9237 and NI-9234 modules. In the meantime, displacement and acceleration responses were also measured, using static (DRA-330A, Tokyo Sokki Kenkyujo Co. Ltd., Tokyo, Japan) data loggers and dynamic (iOtech-652U, IOtech, Inc., Norton, MA, USA) data loggers under the wired setting. Figure 6 presents a modal shaker (EDS50-120, Famtech Co. Ltd., Changwon, Korea) and also wired and wireless data loggers. Figure 6a shows particularly the WUMS installed to acquire wireless responses (displacement, acceleration) while Figure 6b shows a dynamic data logger needed to get wired displacement responses. Figure 6c illustrates a dynamic data logger installed to obtain wired acceleration responses. To measure displacement responses, LVDT (CDP-50, Tokyo Sokki Kenkyujo Co. Ltd., Tokyo, Japan) was used. An accelerometer (3134D, Dytran Instrument, Inc., Chatsworth, CA, USA) was also adopted to measure acceleration responses.

**Figure 7:**
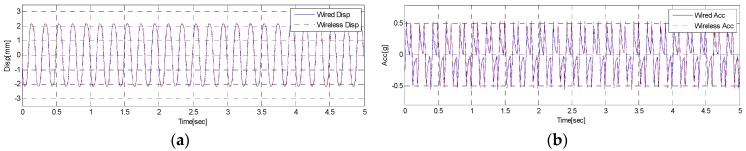
Wired and wireless response using a modal shaker: (**a**) Comparison of displacement; (**b**) comparison of acceleration.

In the test, static and dynamic responses were obtained from the modal shaker (5 Hz, sine wave) through wired and wireless communications. The wired and wireless responses from the response test were shown in Figure 7. Figure 7a shows displacement responses obtained through wired and wireless communications while Figure 7b states acceleration responses acquired through wired and wireless communications. As shown in Figure 7, both results are found to be nearly the same, whether wired or wireless. It is thus found that the WUMS developed in this study is good enough to be a data logger without being wired, and successfully measure multiple responses through wireless communication simultaneously.

### 3.3. Modal Test on a Model Bridge

**Figure 8:**
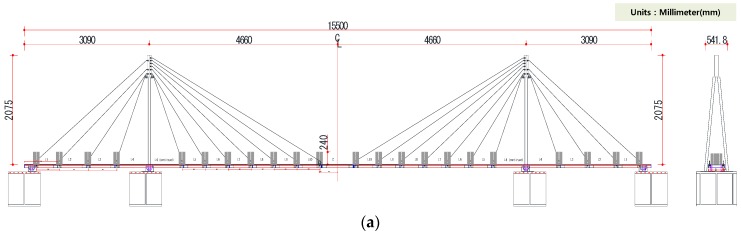

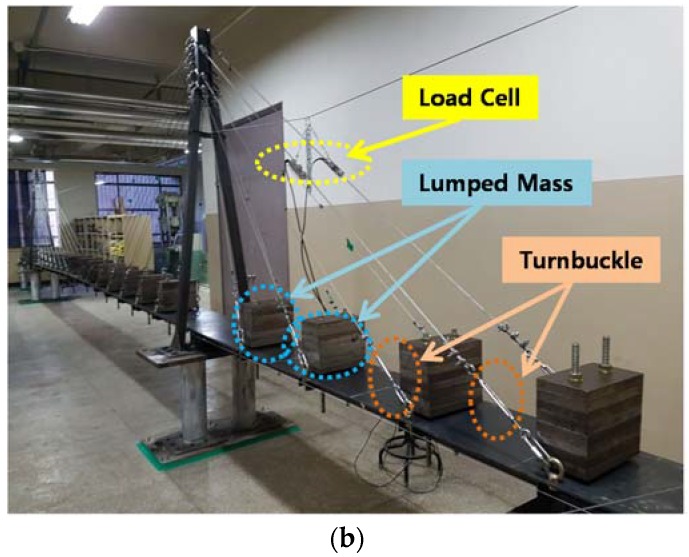
Model bridge: (**a**) Design of model bridge; (**b**) product of model bridge.

To check the structural response performance of the WUMS and examine its practicality, a modal test was performed, using a model bridge. For a model bridge, a Korean flagship cable-stayed bridge called ‘Dolsandaegyo Bridge’ was reduced in size to 1/30 scale and designed as shown in Figure 8a and actually built as illustrated in Figure 8b.

As a model bridge, we built a cable-stayed bridge whose total length is 15,500 mm, with a middle span of 9320 mm and a side span of 3090 mm. Its tower was as high as 2075 mm while its width 400 mm. Forty-four cables in total were used to sustain the load of superstructure. In addition, a lumped mass of 80 kg was applied to 23 spots (22 cable spots, 1 middle spot in the middle span) to have the model bridge experience relatively low natural frequency. All bridge structures but cables were designed with common steel. For cables, stainless steel wires (7 × 19 strand) of 4 mm and 6 mm in diameter were adopted. To impose the constraint conditions on the model bridge, a tower hinge was used on the left, and rollers were adopted on both right and left sides. Lastly, each cable was tied symmetrically from the inside to the outside based on the main tower in accordance with the tension introduction of the actual bridge. To facilitate a smooth introduction of tension, this study adopted a turnbuckle whose loosening and tightening of the turnbuckle were to generate tension, which was confirmed by means of a tension-measuring load cell (Model WLFN2 Series) specifically used for a 500 kg rope.

**Figure 9:**
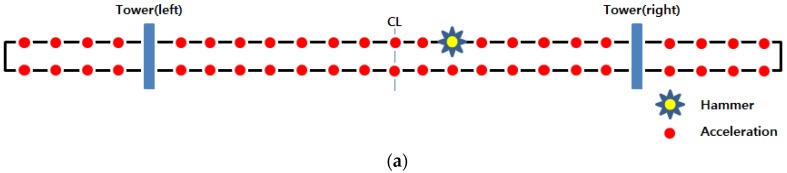

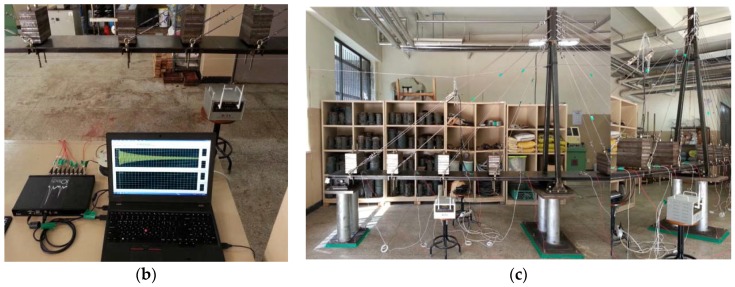
Modal test of model bridge: (**a**) Hammer and sensor location; (**b**) wired measurement system; (**c**) wireless measurement system (WUMS).

As shown in Figure 9a, a modal test in this study was performed after selecting a total of 46 acceleration response measurement spots and a single hammer position on the model bridge. To measure structural hammering, an impact hammer (PCB-086D20) was used. For the measurement of acceleration responses, a commercial accelerometer (Dytran-3134D, Chatsworth, CA, USA) was applied. In each measurement spot, 256 data per second were obtained, using the wired data logger (iOtech-652U) as in Figure 9b and the wireless WUMS as in Figure 9c. Then, acceleration responses were acquired for about 30 s.

**Figure 10:**
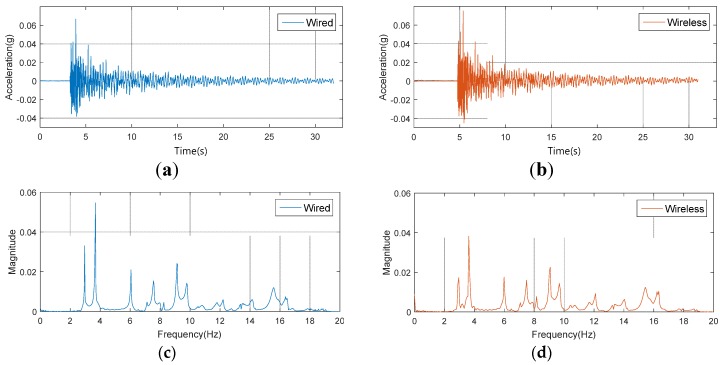
Results of modal test: (**a**) Wired time domain; (**b**) wireless time domain; (**c**) wired frequency domain; (**d**) wireless frequency domain.

Figure 10 shows both wired and wireless acceleration response signals which were obtained through the modal test in time and frequency domain. In both time and frequency domains, the wired and wireless acceleration responses were mostly matched as shown in Figure 10. As illustrated in Figure 10, this study calculated the frequency response function (FRF) of the model bridge, using acceleration response signals at each measurement point (64 points in total). Then, the modal parameters (natural frequency, mode vector) of the model bridge were calculated through modal analysis. From Figure 10c,d, the magnitudes of the first and two peaks (first and second bending mode) from wireless signals are relatively lower than those from the wired signal. These two mutually compared responses (wired and wireless) were not obtained at the same time. These errors are judged by the experimental error due to the moving installation of a number of acceleration sensors (total 46 acceleration measurement points) and the strength of the impact hammering.

In order to compare modal parameters (natural frequency, mode vector) obtained by each of the wired and wireless responses, this study considered four bending modes and two torsion modes.

First, Table 3 shows a comparison of the natural frequency of the model bridge obtained through modal analysis in wired communication with that of wireless one. As stated in this table, natural frequency revealed 2.7% mean error in wireless communication. After all, wireless natural frequency and wired natural frequency was matched to a significant degree in four bending modes and two torsion modes. The structural responses obtained through wireless communication sufficiently reflected the dynamic characteristics of the model bridge.

**Figure 11:**
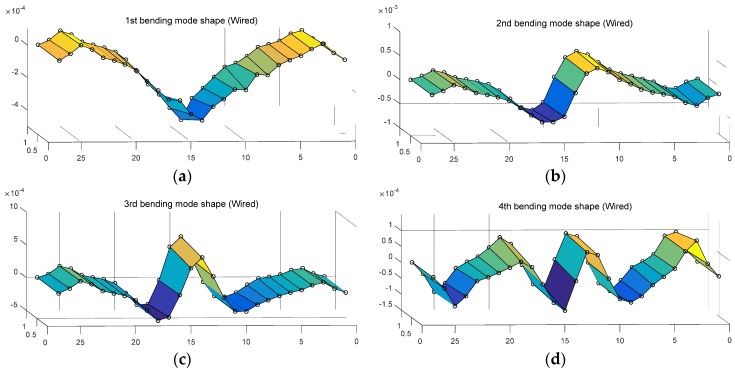

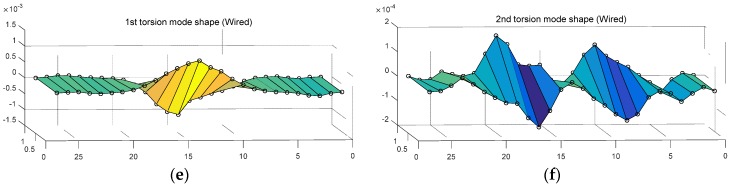
Wired mode shape: (**a**) 1st bending mode; (**b**) 2nd bending mode; (**c**) 3rd bending mode; (**d**) 4th bending mode; (**e**) 1st torsion mode; (**f**) 2nd torsion mode.

**Figure 12:**
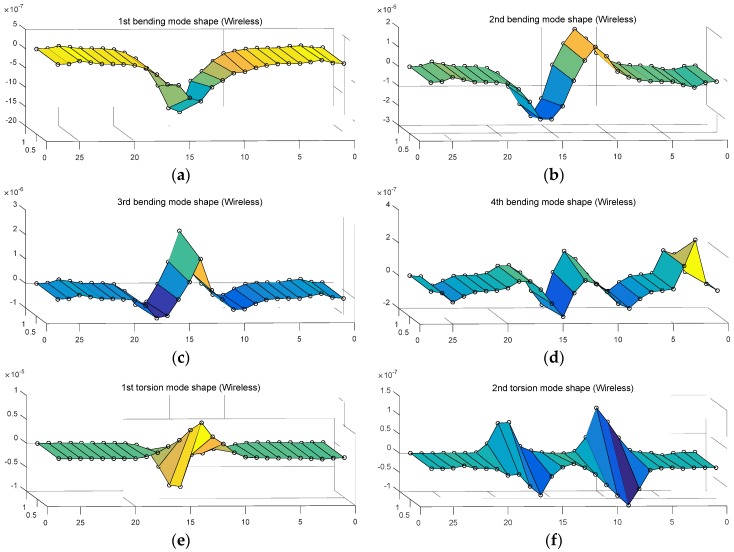
Wireless mode shape: (**a**) 1st bending mode; (**b**) 2nd bending mode; (**c**) 3rd bending mode; (**d**) 4th bending mode; (**e**) 1st torsion mode; (**f**) 2nd torsion mode.

Then, in order to assess the structural responses obtained through wireless communication, this study estimated mode vector through modal analysis as well as the natural frequency of the model bridge as stated in Table 3. Figure 11 and Figure 12 show the mode shapes on four bending modes and two torsion modes respectively, using the mode vector obtained from the wired and wireless responses. In Figure 11 and Figure 12, the 6 modes revealed the typical bending and torsion of cable-stayed bridge. The mode shape from wireless responses in Figure 12 showed similar behavior, compared to the mode shape from wired responses in Figure 11. This study assessed modal assurance criteria (MAC) on the mode shape of a total of six wired and wireless responses from Equation (1) as in Figure 11 and Figure 12 [25].

(1) $$\mathrm{MAC}\;\left(\mathrm{wired},\mathrm{wireless}\right)=\frac{{\left|\sum_{j=1}^{n}{\left(\emptyset_{wireless}\right)}_{j}{\left(\emptyset_{wired}\right)}_{j}^{T}\right|}^{2}}{\left(\sum_{j=1}^{n}{\left(\emptyset_{wireless}\right)}_{j}{\left(\emptyset_{wireless}\right)}_{j}^{T}\right)\left(\sum_{j=1}^{n}{\left(\emptyset_{wired}\right)}_{j}{\left(\emptyset_{wired}\right)}_{j}^{T}\right)}$$

Here, $\emptyset_{wireless}$ and $\emptyset_{wired}$ represent the mode vectors obtained from wireless and wired responses respectively. If the two modes are the same, the MAC is ‘1’. If they are not related with each other at all, MAC is ‘0’ [22]. Ewins [22] said that even though MAC is usually within 0.9 in regard to the correlated modes, it could permit up to 0.7 depending on circumstances.

Table 4 states modal correlation on six objective modes calculated using Equation (1) in MAC. From Table 4, modal correlation was found (0.8 or higher in MAC) in six objective modes. After all, the mode vectors obtained through wireless response by objective mode revealed excellent modal correlation (bold numbers) compared to the mode vector obtained from the wired responses. Along with natural frequency, they were successfully able to reflect the dynamic characteristics of model bridge.

## 4. Conclusions

In this study, we developed the WUMS as a SHM and successfully tested its performances, proving its validity by means of the following tests: (i) A wireless communication distance test in an open field, (ii) a response test using a modal shaker, (iii) a prototype bridge-based modal test. The followings are concluded from the test results:

(1) According to the wireless communication distance test in an open field, the WUMS was able to graps signals far up to 500 m at WiFi 2.4 GHz and also up to 800 m at WiFi 5.0 GHz. After all, the WUMS is proven to perform a SHM using the wireless technology at a short range (within 500 m) even with obstacles at WiFi 2.4 GHz. It is also found that it can work as a SHM using the wireless technology in the distance (within 800 m) when linearity is relatively guaranteed without any obstacles at WiFi 5 GHz.
(2) From the response test using a modal shaker, the WUMS was able to acquire multiple responses (e.g., acceleration, displacement, strain, etc.) simultaneously using the wireless communication through a single data logger. The acquired wireless responses were matched to a large degree with wired responses so that they were valid for wireless data loggers for a SHM.
(3) According to the model bridge-based modal test, the WUMS was able to get valid dynamic responses with 0.8 or higher in modal correlation with about 2.7% error rates of natural frequency compared to wired responses in realtime.
(4) In addition, the WUMS developed in this study has its own power source for nearly 48 hours with a Li-ion battery. In addition, the adoption of the FPGA-based high-performance controller, which provides a three slotted I/O, enables a measurement of multi-channel responses when the same measurement models are used as well as the measurement of multiple responses including GPS. Since the FPGA programming is available, the system could be operated in standalone mode in a stable manner on the basis of RTOS.
(5) There will be further studies on practicality by applying the WUMS to real structures. In addition, the studies on SHM will continue, specially using the structural responses wirelessly obtained in realtime such as GPS information and multiple responses.

## Figures and Tables

**Table 1:** Wireless communication distance and receive sensitivity at WiFi 2.4 GHz.

2.4 GHz	100 m	200 m	300 m	400 m	500 m	600 m	700 m	800 m	900 m	1 km
Test 1	89%	83%	77%	74%	74%	-	-	-	-	-
Test 2	89%	79%	75%	73%	72%	-	-	-	-	-
Test 3	86%	75%	75%	71%	68%	-	-	-	-	-
Test 4	89%	75%	75%	71%	70%	-	-	-	-	-
Test 5	85%	77%	74%	73%	70%	-	-	-	-	-
Wirelss Comm.	O	O	O	O	O	X	X	X	X	X
	Where “O” and “X” indicate the success and failure of wireless communication.									

**Table 2:** Wireless communication distance and receive sensitivity at WiFi 5 GHz.

5 GHz	100 m	200 m	300 m	400 m	500 m	600 m	700 m	800 m	900 m	1 km
Test 1	100%	80%	67%	64%	64%	60%	57%	70%	-	-
Test 2	98%	77%	68%	62%	62%	61%	60%	60%	-	-
Test 3	98%	90%	67%	64%	64%	62%	62%	60%	-	-
Test 4	95%	80%	66%	62%	62%	58%	57%	60%	-	-
Test 5	99%	78%	68%	63%	63%	62%	61%	61%	-	-
Wireless Comm.	O	O	O	O	O	O	O	O	X	X
	Where “O” and “X” indicate the success and failure of wireless communication.									

**Table 3:** Compare to natural frequency of model bridge (wired vs. wireless).

Mode	Wired (Hz)	Wireless (Hz)	Error (%)
1st Bending	2.94	3.02	2.721
2nd Bending	3.63	3.75	3.305
3rd Bending	6.01	6.16	2.495
4th Bending	7.05	7.22	2.411
1st Torsion	8.21	8.41	2.436
2nd Torsion	10.5	10.8	2.857
Average			2.704

**Table 4:** Compare to mode vector of model bridge (wired vs. wireless).

	Wired	1st Bending	2nd Bending	3rd Bending	4th Bending	1st Torsion	2nd Torsion
Wireless							
1st Bending		**0.8780**	0.0245	0.0746	0.0048	0.0208	0.0001
2nd Bending		0.0288	**0.9193**	0.0068	0.0218	0.0011	0.0013
3rd Bending		0.1097	0.0036	**0.8572**	0.0029	0.0460	0.0015
4th Bending		0.0125	0.0320	0.0587	**0.8981**	0.0010	0.0007
1st Torsion		0.0030	0.0026	0.0014	0.0194	**0.8931**	0.0259
2nd Torsion		0.0002	0.0025	0.0001	0.0037	0.0036	**0.8211**

## Abbreviations

The following abbreviations are used in this manuscript:

SHM	Structural Health Monitoring
WUMS	Wireless Unified-Maintenance System
EST	Embedded Software Technology
FPGA	Field Programmable Gate Array
RTOS	Real-Time Operating System
GUI	Graphical User Interface
FIFO	First-in First-out
DAQ	Data Acquisition
RF	Radio Frequency
AP	Access Point
I/O	Input and Output
FRF	Frequency Response Function
MAC	Modal Assurance Criteria
